# Early Intranasal Vasopressin Administration Impairs Partner Preference in Adult Male Prairie Voles (*Microtus ochrogaster*)

**DOI:** 10.3389/fendo.2017.00145

**Published:** 2017-06-28

**Authors:** Trenton C. Simmons, Jessica F. Balland, Janeet Dhauna, Sang Yun Yang, Jason L. Traina, Jessica Vazquez, Karen L. Bales

**Affiliations:** ^1^Department of Psychology, University of California Davis, Davis, CA, United States

**Keywords:** pair-bond, fecal boli, play, anxiety, social, aggression

## Abstract

Research supports a modulatory role for arginine vasopressin (AVP) in the expression of socially motivated behaviors in mammals. The acute effects of AVP administration are demonstrably pro-social across species, providing the justification for an ever-increasing measure of clinical interest over the last decade. Combining these results with non-invasive intranasal delivery results in an attractive system for offering intranasal AVP (IN-AVP) as a therapeutic for the social impairments of children with autism spectrum disorder. But, very little is known about the long-term effects of IN-AVP during early development. In this experiment, we explored whether a single week of early juvenile administration of IN-AVP (low = 0.05 IU/kg, medium = 0.5 IU/kg, high = 5.0 IU/kg) could impact behavior across life in prairie voles. We found increases in fecal boli production during open field and novel object recognition testing for the medium dose in both males and females. Medium-dose females also had significantly more play bouts than control when exposed to novel conspecifics during the juvenile period. Following sexual maturity, the medium and high doses of IN-AVP blocked partner preference formation in males, while no such impairment was found for any of the experimental groups in females. Finally, the high-dose selectively increased adult male aggression with novel conspecifics, but only after extended cohabitation with a mate. Our findings confirm that a single week of early IN-AVP treatment can have organizational effects on behavior across life in prairie voles. Specifically, the impairments in pair-bonding behavior experienced by male prairie voles should raise caution when the prosocial effects of acute IN-AVP demonstrated in other studies are extrapolated to long-term treatment.

## Introduction

Arginine vasopressin (AVP) is a neuropeptide, which exerts its effects in both the brain and periphery. Within the brain, the AVP system acts to influence socially motivated behaviors (1) utilizing several different neurocircuits (2), including social recognition, communication, and aggression. The AVP system is widespread throughout the central nervous system well before birth (3), suggesting an organizational role in development (4).

Early manipulations of the AVP system have been shown to alter behavior across life. In rats, prenatal AVP injections impact fetal suckling behavior (5) while juvenile injections of AVP receptor 1a antagonists disrupt play behavior (6). The effects of early postnatal injections of AVP can stretch into adulthood, increasing male aggression in prairie voles (7) and affiliative attachment in zebra finches (8). Pharmacological manipulations of the AVP system help elucidate its various functions while confirming the presence of critical periods for the organizational impact of AVP signaling.

More recently, studies have found associations between disruption of the AVP system and the expression of certain neurodevelopmental disorders in humans, like autism spectrum disorder (ASD). For example, plasma AVP levels [Ref. (9–11); but see Ref. (12)] and certain single-nucleotide polymorphisms of the genes for AVP and its receptor (13) have been correlated with social functioning in individuals with ASD. Given the frequently pro-social effects of acute intranasal AVP (IN-AVP) administration in humans (14–16) and animal models (17, 18), IN-AVP has been suggested as a treatment for the social deficits in children with ASD (19, 20).

However, acute studies have left several important questions unanswered. Specifically, do the potentially beneficial aspects of acute administration extend to chronic administration? Or, could prolonged exposure cause unforeseen long-term effects? Thus, the purpose of our study was to explore the long-term effects of early IN-AVP administration on behavior across life in prairie voles (*Microtus ochrogaster*). We administered three doses of AVP (low = 0.05 IU/kg, medium = 0.5 IU/kg, high = 5.0 IU/kg) or saline twice daily to male and female prairie voles from age 15 to 21 days. This age range falls within the early juvenile period in prairie voles, approximating the developmental stage at which children are being treated with IN-AVP in at least one clinical trial (https://ClinicalTrials.gov Identifier: NCT01962870). The medium dose reflects the dose used in these trials but controlled for weight. As voles are typically weaned around day 20, we explored whether parental behavior changed because of pup treatment, and then, each animal postweaning was tested in several experimental paradigms. From tests of anxiety, exploration, and sociality in the juvenile period to tests of partner preference formation and aggression in adulthood, we explored whether a single week of IN-AVP exposure could perpetuate behavioral changes across life.

We hypothesized that the effects of IN-AVP would vary by dose, possibly representing differential activation of multiple AVP sub-circuits within the brain, or activation of oxytocin receptors at high doses. Male prairie voles have higher AVP immunoreactivity in several brain regions, including the lateral septum, lateral habenular nucleus, and bed nucleus of the stria terminalis [Ref. (21, 22); but see Ref. (23)]. As such, we expected IN-AVP to have the most profound effects in males. Finally, we predicted that the effects of IN-AVP would be context-specific, increasing sociality during non-threatening encounters (e.g., juvenile affiliation) and increasing aggression during competitive encounters (e.g., adult affiliation following pair-bond formation).

## Materials and Methods

### Subjects

We recruited 103 prairie vole subjects (52 males, 51 females) from our breeding colony located in the Department of Psychology at the University of California, Davis. We maintained the animals on a 14:10 h light cycle at approximately 21°C and provided food (Purina High Fiber Rabbit Chow, PMI Nutrition International, Brentwood, MO, USA) and water *ad libitum*. Animals were housed in large polycarbonate cages (44 cm × 22 cm × 16 cm) with their parents and marked with non-toxic Nyanzol D dye (American Color and Chemical Corporation, Charlotte, NC, USA) for identification purposes until weaning at postnatal day (P) 20. We then separated all subjects from their parents, gave them ear clip markings, and placed them with a same-sex sibling in smaller cages (27 cm × 16 cm × 13 cm) until sacrifice. Subjects that were treated with IN-AVP were housed with untreated siblings. To help control for potential litter effects, each litter had at least one AVP-treated animal and one saline-treated animal within sex.

### Intranasal Treatments

Each test subject was randomly assigned to one out of four treatment groups, including saline control, low-dose AVP (0.05 IU/kg), medium-dose AVP (0.5 IU/kg), and high-dose AVP (5.0 IU/kg). The medium dose was specifically calculated to represent the same dose given in some clinical trials (https://ClinicalTrials.gov Identifier: NCT01962870), only controlled for weight. AVP solutions were purchased from Sigma-Aldrich (V0377 SIGMA), already mixed in NaCl. The solution was then diluted to provide the necessary concentrations for the treatment groups and aliquoted into 200 µL test tubes. The tubes were stored in a refrigerator at 4°C until use.

From P15 to 21 (early juvenile period), voles were given intranasal treatments twice daily. Each day, the first treatment was given between 0900 and 1100 hours, while the second treatment was given between 1500 and 1800 hours. Treatments were administered through cannula tubing, which was attached to a blunt cannula needle (33 gauge, 2.8 mm length; Plastics One, Roanoke, VA, USA) secured to an airtight Hamilton syringe (Fisher Scientific, Pittsburgh, PA, USA). The animal was held still while 25 µL of solution was expelled slowly through the cannula system and allowed to absorb into the nasal mucosa (divided evenly between the two nostrils). Following administration, the animal was returned to its home cage while the Hamilton syringes and cannula system were cleaned with 70% isopropyl alcohol solution and de-ionized water. Treatment order was randomized each day and administration was rapid (less than 30 s) making handling consistent across treatment groups.

### Observations and Behavioral Testing

**Figure 1:**
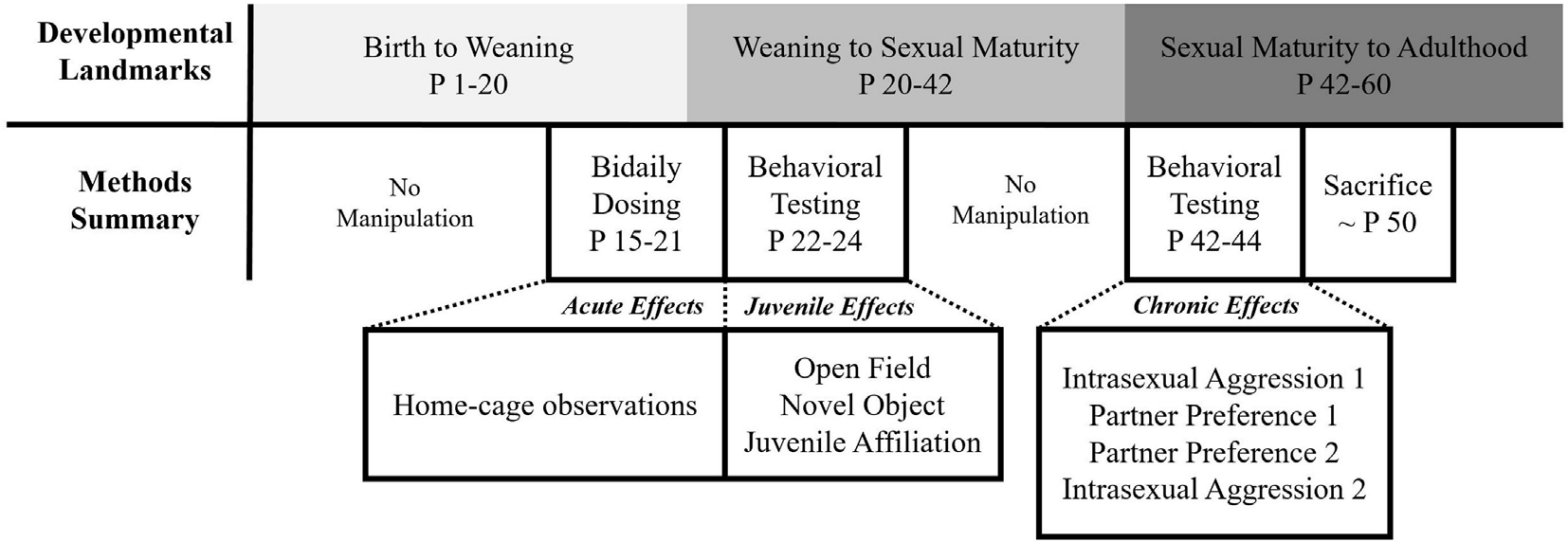
Summary of experimental procedures.

All animals were subjected to a series of testing paradigms from weaning to adulthood. These tests were digitally recorded and manually scored using Behavior Tracker 1.5 (www.behaviortracker.com). Each scorer was blind to subject group assignment. See Figure 1 for a summary of all experimental procedures.

#### Acute Observations

Following the first treatment on the first (P15) and sixth day (P20) of dosing, each animal was observed in their home-cage approximately 15 min posttreatment for a total of 5 min. We measured the duration of contact, nursing, and licking/grooming behaviors directed toward the subjects.

#### Open Field Test

All subjects received an open field test on P22. The open field consisted of a 40 cm × 40 cm × 40 cm Plexiglas box with a 5 × 5 grid marked on the floor. At the beginning of the test, the vole was placed in the center of the arena while their behavior was digitally recorded for the following 10 min using a camera. Observers recorded the frequency of line crosses, fecal droppings, and rearing and the duration of autogrooming, thigmotaxis, and freezing behavior.

#### Novel Object Recognition Test

We split the test into two phases, which included a familiarization phase (NOF) and a testing phase (NOT) occurring on separate, consecutive days following open field testing (P23 and 24, respectively). Both phases were conducted in the open field test arena to help habituate the animals to the environment. Thus, subtle differences in object interactions would not be overshadowed by the environment’s novelty.

During the familiarization phase, two identical objects were placed in opposite corners of the open field arena. Like open field testing, the subject was placed in the center of the arena and their behavior was recorded for 10 min. The following day, the animal was reintroduced to the arena for the testing phase for 10 more minutes. During this phase, a familiar object from the day before was placed into the arena with a novel object; object placement was consistent between the tests. We measured the same behaviors in this paradigm as open field while including a measure for the duration of time spent interacting with the objects.

#### Juvenile Affiliation Test

At P25, each test subject and novel conspecific was placed into a neutral cage (27 cm × 16 cm × 13 cm) where interactions were digitally recorded for 10 min. Observers recorded affiliative behaviors (sniffing, contact, allogrooming, play), anxiety-related behaviors (digging, rearing, autogrooming, defensive rearing), and aggressive behaviors (i.e., lunging, chasing, wrestling). Play behavior was recorded as a sum of the different behaviors described by Chau et al. (24). In addition to play and rearing, all aggressive behaviors were recorded as frequencies. All other behaviors were recorded as durations.

#### Intrasexual Aggression Tests

The intrasexual aggression test was similar to the juvenile affiliation test, with two significant differences: (1) the stimulus animal was collared for identification purposes, (2) the test was done twice during adulthood. The first test occurred the day before partner preference testing (P42) and the second took place the day after (P45). The first test provided a baseline for adult sociality and aggressiveness while the second was meant to test for post-pair-bonding behaviors like mate-guarding. Each test was digitally recorded for 10 min and observers scored all the same behaviors as in juvenile affiliation.

#### Partner Preference Tests

Following the first intrasexual aggression test, all subjects underwent two partner preference tests over two consecutive days (P43 and 44, respectively). For partner preference testing, each test subject was given a cohabitation period with a sexually naïve partner of the opposite sex (25). For the first partner preference test, male subjects underwent a cohabitation of 2 h, while females were given 30 min. The discrepancy in cohabitation times between the sexes reflect differences in the time it takes for males and females to form a pair-bond naturally. While females can form a pair-bond after only 6 h of cohabitation, males generally require at least 24 h (26). Thus, the deficient cohabitation periods employed on the first day were meant to test for IN-AVP-stimulated facilitations of pair bonding.

Following the cohabitation period, the partner and an additional mate choice (“stranger”) were loosely tethered within distinct testing chambers. Tethers consisted of a cable tie around the neck of the vole (employed carefully while animals are monitored) attached to fishing line, which is then secured firmly to the side of the cage. The testing apparatus consisted of three identical polycarbonate cages (27 cm × 16 cm × 13 cm) attached by Plexiglas tubes (8.5 cm × 16 cm). The test animal was free to move throughout the apparatus while the two stimulus animals were confined to their separate chambers. The three-chambered paradigm provided the subjects with a choice of a familiar partner, novel stranger, or an empty cage for 3 h. Food and water was readily available in all chambers throughout the testing period.

Following the first partner preference test, the test animal and familiar partner were housed together overnight. A second partner preference test was then done the following day (approximately 24 h of cohabitation between tests) after a sufficient cohabitation period was provided to normally establish a pair-bond in both males and females. Thus, the second partner preference test was used to detect potential IN-AVP-stimulated deficits in pair bonding. A different stranger vole was used for this second test. For both tests, we measured the duration of cage location and side-to-side contact while recording the frequency of aggression.

#### Weight

To determine whether the potential effects of IN-AVP administration could be explained by weight changes, we measured all subjects on the first day of treatment, last day of treatment, and once after all testing had been completed.

### Statistical Analyses

As direct treatment comparisons across the sexes were confounded by the difference in behavioral baselines, we decided to analyze males and females separately. Thus, we examined the effects of developmental AVP exposure in both sexes but not between sexes. We also controlled for the potentially confounding effect of litter on our results by assigning a unique identifier to all pups from the same litter and including this variable in our analyses.

All analyses were conducted using R version 3.3.3 (27). We began by fitting two models for each dependent variable, one including the litter variable as a random effect and one without it; both models included treatment group as a fixed effect. These two models were compared using an exact likelihood ratio test from the RLRsim package (28) to determine whether the presence of the variance component provided a better fit for the model. The test statistic from this likelihood ratio test is based on simulated values from the exact sample distribution as derived by Crainiceanu and Ruppert (29). When the statistic of the observed likelihood ratio was significant (α = 0.05), we chose the mixed model over the linear model, having found evidence for a significant effect of litter on the dependent variable. Each model that included the random effect was fit using the lme4 package (30), while all other models were fit using base R functionality.

After selecting the best model, we conducted a series of follow-up tests to confirm that our model met the assumptions for ANOVA testing. For the normality assumption, we prioritized visual inspection of Q–Q plots (31), but confirmed our observations using a combination of the Shapiro–Wilk test and measures of skewness and kurtosis. Despite the Shapiro–Wilk test having the best power for a given significance when compared to other normality tests (32), it is biased by sample size (33). Therefore, normality was assumed when the Shapiro–Wilk test was statistically insignificant (α = 0.05), the Shapiro–Wilk test statistic was high (*W* > 0.95), or when values for skewness and kurtosis fell between −2 and +2 (34, 35). We also utilized Levene’s test to determine whether group variances were homogenous. When models contained outliers or heteroscedastic data, we refit the model using robust techniques (36, 37). Robust linear models were fit using the MASS package (38), while robust mixed linear models were fit using the robustlmm package (39).

After selecting the best model for each dependent variable and satisfying the assumptions for one-way ANOVA testing, we passed the models to the car package (40) to produce the ANOVA tables. For mixed ANOVA models, *F*-test statistics were calculated using Kenward–Roger’s approximation for degrees of freedom. Using the lsmeans package (41), we conducted *post hoc* analyses on all models that contained a statistically significant effect of treatment (α = 0.05). We only considered direct comparisons between each treatment group and control, warranting the use of Dunnett’s test to control for Type I errors (42).

To determine whether overall parental handling differed between the groups, we combined the data from the dam with the sire and then compared total parental handling on individual observation days. Preliminary analyses confirmed no treatment differences within each observation day, so we then summed all parental handling behaviors across both days and reanalyzed the data. For partner preference data, we standardized the contact scores by subtracting the time spent with the stranger from the time spent with the partner, depicting the magnitude of the preference for the partner over the stranger. We tested whether our difference scores were significantly greater than 0, indicating a preference for the partner over the stranger. Then, we compared these scores across treatment groups to see if the magnitude of partner preference was affected by AVP treatment.

## Results

### Early Effects

Intranasal AVP administration had no effect on acute parental handling; Table 1. For the open field test, IN-AVP altered fecal boli production in males, *F* (3, 51) = 2.839, *p* < 0.05. Specifically, males treated with the medium dose produced more fecal boli than control, *z* = 2.801, *p* < 0.05. IN-AVP also altered fecal boli production in females, *F* (3, 50) = 4.497, *p* < 0.01; high-dose exposure increased fecal boli production relative to control, *z* = 3.650, *p* < 0.001. We did not find effects of IN-AVP on any other recorded behavior in either sex; Table 2.

**Table 1:** Parental handling and weight change statistics.

		Acute observations	Weight
Sex	Group	Parental handling	Weight change
Males	Control	446.0 ± 47.7	25.3 ± 1.0
	Low	408.8 ± 82.8	29.1 ± 1.5
	Medium	528.4 ± 98.7	27.0 ± 1.7
	High	434.4 ± 84.3	24.9 ± 1.1

Females	Control	423.5 ± 37.6	19.5 ± 0.8
	Low	425.2 ± 48.7	21.0 ± 1.2
	Medium	373.5 ± 52.0	19.9 ± 1.7
	High	290.5 ± 56.0	20.6 ± 1.8

*Values represent empirical means ± SEM. Parental handling represents the total amount of parental contact received (e.g., licking, nursing) across two individual observations periods (seconds). Weight change represents the weight gain from day 1 of treatment to sacrifice (grams)*.

**Table 2:** Arena test statistics.

	Sex	Group	Line crosses	Autogrooming	Rearing	Exploration	Fecal boli	Freezing
Open field	Males	Control	475.3 ± 58.8	26.0 ± 5.7	41.7 ± 6.0	64.7 ± 9.3	1.7 ± 0.6	31.1 ± 4.2
		Low	370.7 ± 67.4	34.7 ± 8.8	40.7 ± 7.0	96.8 ± 28.2	2.4 ± 1.0	40.7 ± 13.4
		Medium	374.5 ± 51.2	21.8 ± 4.7	41.3 ± 9.5	60.7 ± 14.6	4.5 ± 1.4	88.6 ± 36.1
		High	392.9 ± 87.2	26.9 ± 10.4	46.5 ± 8.7	61.8 ± 15.8	1.7 ± 0.8	24.5 ± 9.3
	Females	Control	362.6 ± 54.9	37.9 ± 7.5	34.5 ± 5.4	59.1 ± 12.1	1.1 ± 0.3	71.9 ± 25.1
		Low	291.6 ± 37.9	25.5 ± 6.3	31.1 ± 6.2	63.5 ± 11.5	3.3 ± 1.4	57.8 ± 16.6
		Medium	326.8 ± 46.9	44.6 ± 10.9	28.4 ± 5.5	59.7 ± 10.8	3.7 ± 1.3	76.0 ± 74.1
		High	351.9 ± 116.4	26.4 ± 10.3	35.1 ± 13.3	46.9 ± 13.2	4.2 ± 1.1	58.3 ± 38.0

Novel object 1	Males	Control	497.3 ± 62.7	26.7 ± 5.1	42.4 ± 5.6	250.8 ± 17.4	1.5 ± 0.4	27.3 ± 5.1
		Low	381.6 ± 96.9	37.1 ± 9.9	41.4 ± 9.4	191.6 ± 34.8	2.2 ± 1.1	33.4 ± 13.8
		Medium	548.7 ± 102.3	16.7 ± 7.0	39.9 ± 6.3	237.0 ± 21.6	3.1 ± 1.3	18.1 ± 4.9
		High	456.0 ± 97.7	16.1 ± 5.8	45.1 ± 13.0	223.6 ± 37.9	3.3 ± 0.7	16.9 ± 7.5
	Females	Control	391.0 ± 55.6	36.4 ± 8.5	37.6 ± 5.8	225.5 ± 16.1	1.0 ± 0.5	31.5 ± 5.9
		Low	277.6 ± 47.4	45.1 ± 7.8	29.3 ± 7.9	205.5 ± 40.0	1.6 ± 0.9	56.7 ± 11.3
		Medium	280.9 ± 43.5	30.9 ± 9.4	33.2 ± 7.8	196.2 ± 32.7	3.1 ± 1.6	84.5 ± 37.8
		High	369.2 ± 126.8	32.3 ± 9.9	48.7 ± 26.1	179.8 ± 23.2	2.6 ± 1.4	33.0 ± 10.8

Novel object 2	Males	Control	381.3 ± 51.9	33.3 ± 7.6	37.7 ± 5.2	−14.8 ± 35.3	1.8 ± 0.6	51.9 ± 15.7
		Low	349.6 ± 108.7	46.8 ± 16.7	34.7 ± 10.4	−8.2 ± 41.7	0.8 ± 0.3	31.6 ± 13.7
		Medium	525.3 ± 96.7	23.3 ± 5.7	44.8 ± 6.5	23.0 ± 36.1	4.1 ± 1.3	38.0 ± 18.2
		High	433.2 ± 106.2	22.6 ± 5.1	47.0 ± 13.4	58.7 ± 22.3	2.8 ± 0.7	27.1 ± 13.2
	Females	Control	295.7 ± 65.4	33.0 ± 7.4	47.4 ± 8.7	−6.5 ± 20.9	1.4 ± 0.5	28.5 ± 7.1
		Low	417.9 ± 48.0	23.1 ± 4.5	38.6 ± 9.4	53.6 ± 43.8	1.3 ± 0.6	23.9 ± 4.6
		Medium	290.7 ± 70.4	47.3 ± 11.8	30.0 ± 8.1	−49.9 ± 50.8	2.7 ± 1.0	50.0 ± 18.6
		High	371.3 ± 132.1	13.9 ± 2.3	50.2 ± 23.4	64.2 ± 45.8	2.7 ± 1.2	22.0 ± 4.0

*Values represent empirical means ± SEM. Line crosses, rearing, and fecal boli represent count data while autogrooming, exploration, and freezing are measured in seconds. The exploration variable is measured differently across the three paradigms. In the open field test, exploration represents the time spent in the center of the arena. During novel object 1, exploration is the total time spent in object zones. For novel object 2, exploration is the difference in time spent with the novel relative to the familiar object*.

**Figure 2:**
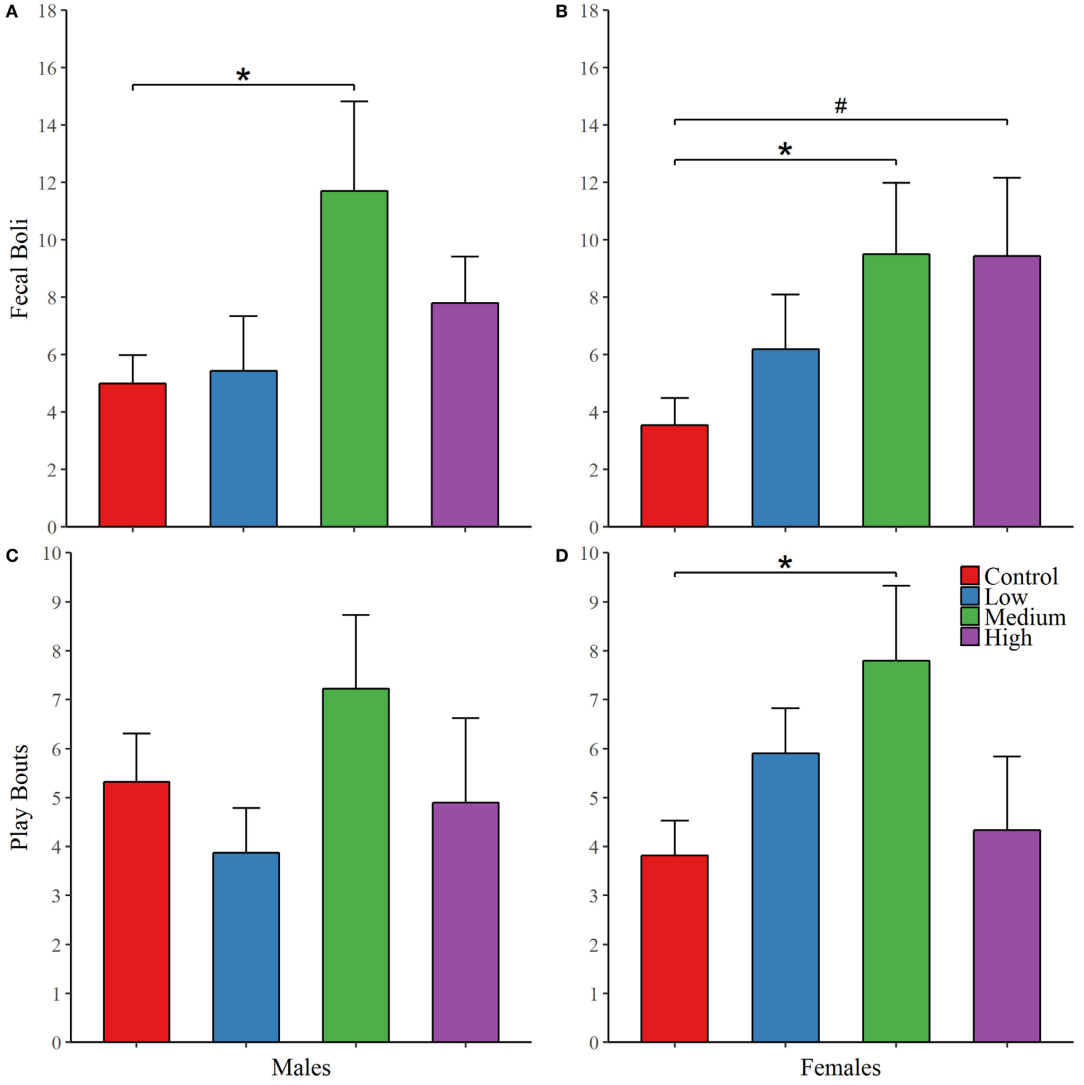
Early exposure to intranasal AVP (IN-AVP) alters juvenile behavior. Values represent group means + SEs. Fecal boli were aggregated across open field and novel object tests (upper row). The dose–response curves appear to differ by sex for fecal boli production; male results reflect a *U*-shaped curve **(A)** and female results approximate a linear effect **(B)**, peaking at the medium dose. Only the medium dose increased fecal boli production in males while both the medium and high doses of IN-AVP increased fecal boli production in females. Bouts of play (bottom row) approximated *U*-shaped curves in both males **(C)** and females **(D)**, only the medium dose in females significantly increased play. *Statistically significant, ^#^trend for significance.

During both phases of the novel object recognition test, the time spent in each object’s interaction zone was similar across treatment groups regardless of sex. In addition, none of the treatment groups, including control, preferentially maintained proximity with the novel object over the familiar object. Like with open field testing, we found no treatment group differences in anxiety or exploratory measures across both phases of recognition testing; Table 2. We decided to combine fecal boli production across the three paradigms to confirm an overall effect of treatment. For males, IN-AVP altered the total fecal boli production across testing days [*F* (3, 51) = 3.656, *p* < 0.05], confirming an increase for medium-dose males (M = 11.70, SEM = 3.13) compared to control (M = 5.00, SEM = 0.99), *z* = 3.036, *p* < 0.01; Figure 2. AVP also impacted the total fecal boli produced across testing days in females, *F* (3, 50) = 3.069, *p* < 0.05; Figure 2. While the high dose (M = 9.44, SEM = 2.71) tended to increase [*t* = 2.39, *p* = 0.056], the medium dose (M = 9.50, SEM = 2.49) significantly increased fecal boli production relative to control (M = 3.54, SEM = 0.95), *t* = 2.502, *p* < 0.05.

For juvenile affiliation, we found no effect of IN-AVP on social- or anxiety-related behaviors in males. But, IN-AVP did impact play behavior in females [*F* (3, 50) = 2.750; *p* = 0.05]; the medium dose increased bouts of play compared to control, *t* = 2.729, *p* < 0.05; Figure 2. No other behaviors were altered by juvenile IN-AVP treatment; Table 3.

**Table 3:** Juvenile affiliation and adult intrasexual aggression test statistics.

	Sex	Group	Sniffing	Autogrooming	Rearing	Play	Aggression
Juvenile affiliation	Males	Control	59.6 ± 6.7	44.7 ± 7.7	41.8 ± 5.2	5.3 ± 1.0	–
		Low	59.4 ± 9.7	57.9 ± 13.4	42.6 ± 7.7	3.9 ± 0.9	–
		Medium	64.1 ± 9.3	43.6 ± 14.5	40.6 ± 5.0	7.2 ± 1.5	–
		High	66.4 ± 7.0	44.0 ± 14.3	36.8 ± 5.6	4.9 ± 1.7	–
	Females	Control	49.6 ± 7.4	37.5 ± 8.0	42.8 ± 7.1	3.8 ± 0.7	–
		Low	101.8 ± 22.7	40.6 ± 11.2	41.7 ± 5.5	5.9 ± 0.9	–
		Medium	92.8 ± 20.9	32.8 ± 11.3	42.5 ± 6.6	7.8 ± 1.5	–
		High	72.7 ± 10.4	45.9 ± 11.4	50.8 ± 9.9	4.3 ± 1.5	–

Intrasexual Aggression 1	Males	Control	84.4 ± 9.9	43.1 ± 7.9	43.8 ± 8.5	–	2.8 ± 1.2
		Low	54.9 ± 11.8	61.2 ± 14.3	33.9 ± 5.2	–	7.0 ± 2.9
		Medium	80.4 ± 14.3	38.2 ± 12.3	42.9 ± 12.0	–	0.7 ± 0.4
		High	93.3 ± 17.0	28.9 ± 6.4	48.3 ± 13.5	–	1.1 ± 0.7
	Females	Control	67.0 ± 9.3	59.5 ± 11.1	47.2 ± 10.1	–	2.0 ± 1.2
		Low	85.1 ± 10.4	60.6 ± 14.9	38.6 ± 4.6	–	0.9 ± 0.5
		Medium	76.7 ± 15.6	52.9 ± 10.4	54.0 ± 10.2	–	3.4 ± 2.6
		High	48.1 ± 10.5	86.0 ± 17.7	45.8 ± 12.8	–	0.9 ± 0.6

Intrasexual Aggression 2	Males	Control	59.6 ± 8.1	76.3 ± 12.0	42.9 ± 7.1	–	9.2 ± 2.4
		Low	37.2 ± 12.5	105.0 ± 27.2	27.3 ± 8.4	–	8.2 ± 6.6
		Medium	56.3 ± 12.0	45.5 ± 23.2	43.3 ± 11.6	–	9.1 ± 6.6
		High	58.6 ± 16.8	65.1 ± 17.9	26.9 ± 5.6	–	20.6 ± 5.5
	Females	Control	49.1 ± 10.1	63.1 ± 14.4	45.9 ± 7.1	–	11.8 ± 2.4
		Low	43.3 ± 15.9	83.7 ± 17.4	44.9 ± 9.8	–	7.0 ± 2.5
		Medium	56.3 ± 19.2	69.9 ± 13.0	49.5 ± 11.3	–	7.9 ± 3.8
		High	61.9 ± 16.2	81.8 ± 26.3	64.1 ± 14.7	–	10.3 ± 4.6

*Values represent empirical means ± SEM. Sniffing and autogrooming are measured in seconds while rearing, play, and aggression are counts*.

### Adult Effects

**Figure 3:**
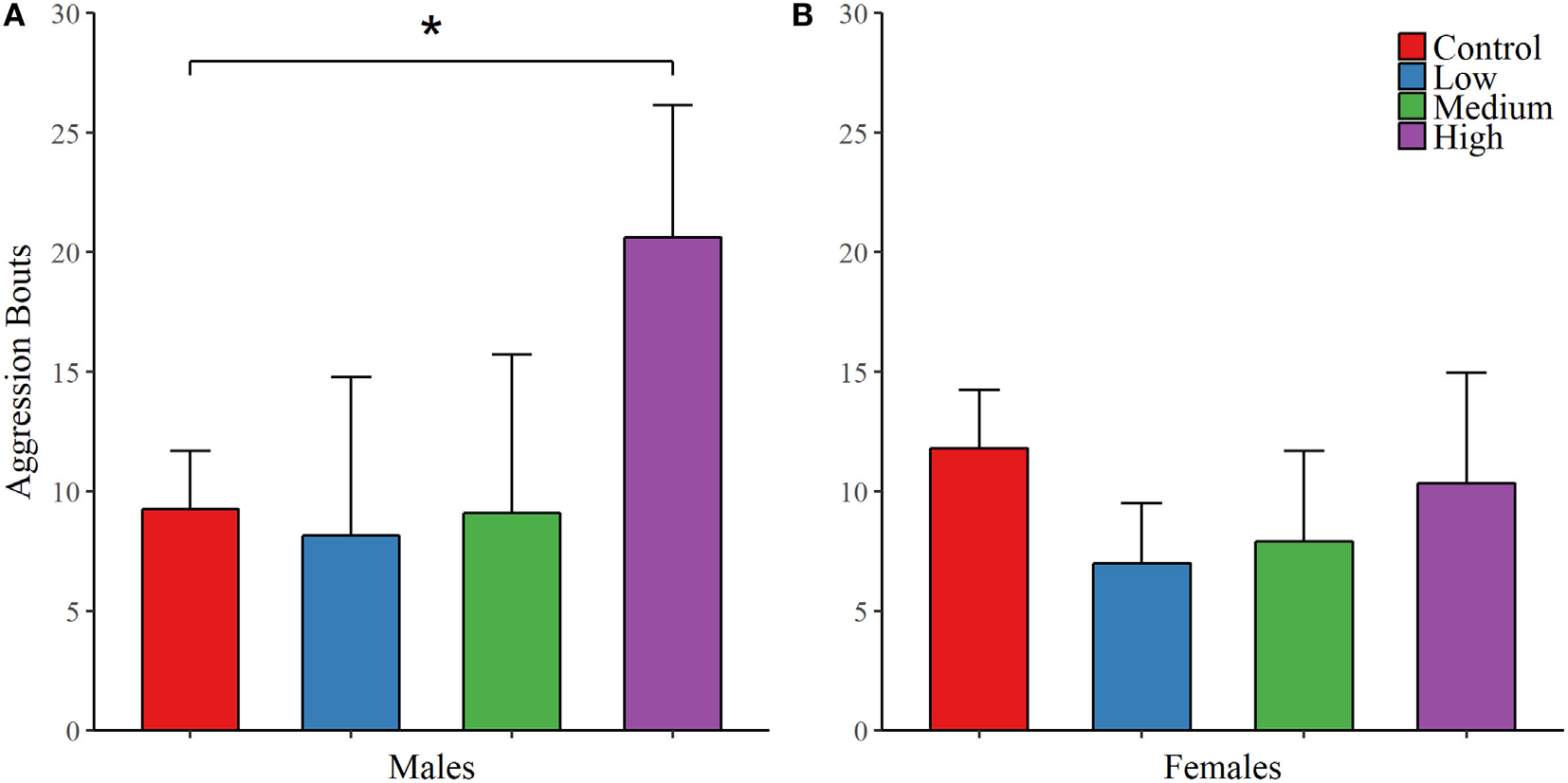
Early exposure to intranasal AVP (IN-AVP) increases aggression in adult males. Values represent group means + SE. While bouts of aggression (e.g., lunging, wrestling) were unchanged during the first iteration of intrasexual aggression testing (not shown in figure), the high dose significantly increased aggression in males following partner preference formation **(A)**. IN-AVP had no detectable effect on female aggression **(B)**.

As with juvenile affiliation, we found no effect of IN-AVP across all recorded behaviors during the first intrasexual aggression test in males; Table 3. However, IN-AVP did have an effect on male aggression during the second intrasexual aggression test, *F* (3, 45) = 4.735, *p* < 0.01. Males treated with the high dose engaged in more bouts of aggressive behavior than control males, *z* = 3.031, *p* < 0.01; Figure 3. For intrasexual aggression testing in females, we found no effect for IN-AVP in females across all recorded behaviors regardless of testing day.

**Figure 4:**
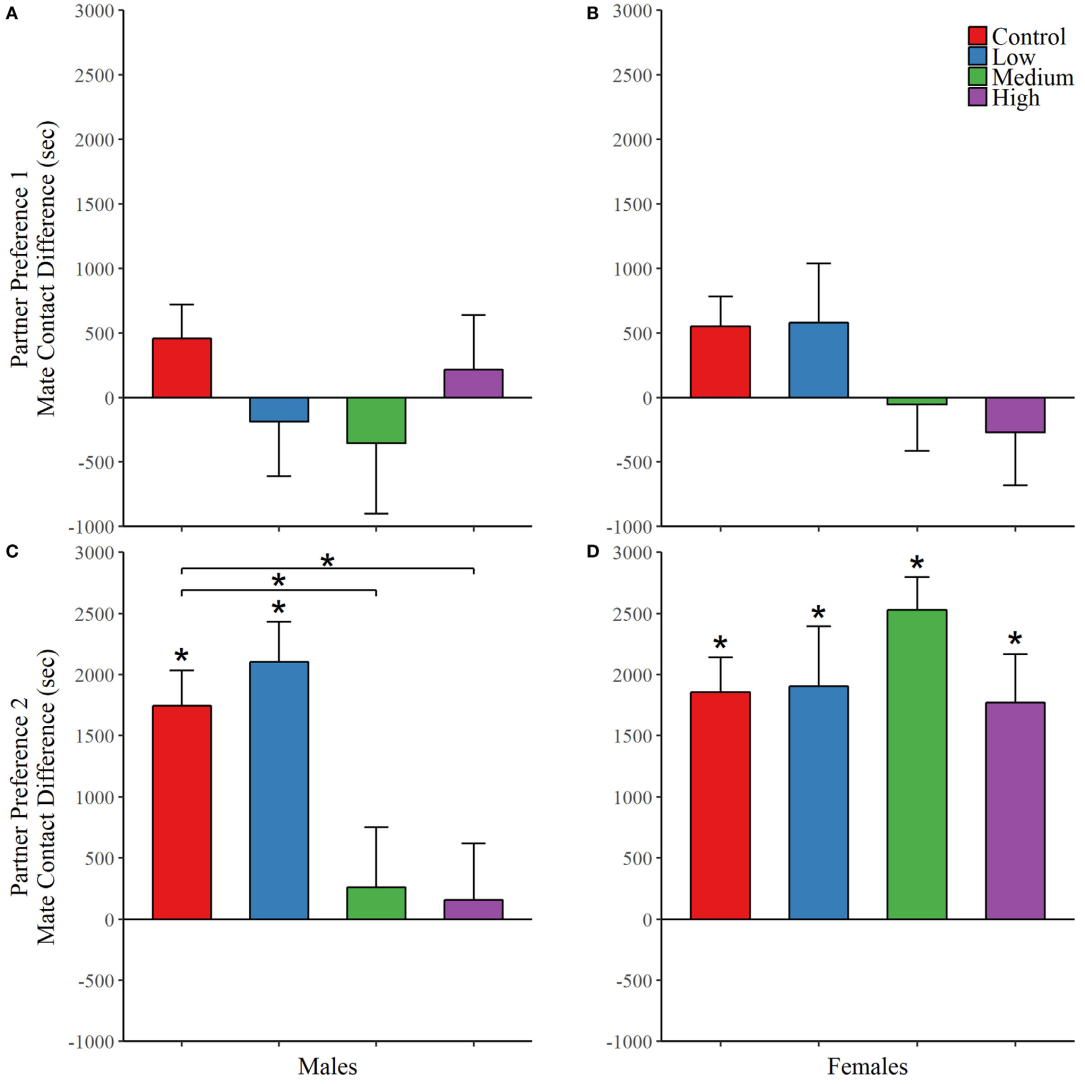
Early exposure to intranasal AVP (IN-AVP) blocks partner preference formation in males. Values represent the mean difference in side-to-side contact between the partner and stranger + SE. Asterisks immediately above group means indicate a significant difference from zero (e.g., more contact with partner than stranger) while asterisks above comparison lines indicate significant group differences in preference. During the first partner preference test, subjects were housed with potential mates for an insufficient amount of time to form a preference (upper row). IN-AVP did not facilitate partner preference in either males **(A)** or females **(B)** during this test. The second partner preference test was completed following 24 h of cohabitation between each test subject and their respective partners from the first test (bottom row). The medium and high doses of IN-AVP shunted partner preference in males **(C)** but all female treatment groups **(D)** successfully preferred the partner over the stranger.

For the first partner preference test, we found no evidence of mate preference for any of the treatment groups; Table 4. However, IN-AVP did have an effect on partner preference in males during the second partner preference test, *F* (3, 50) = 5.847, *p* < 0.01 (Figure 4; Figure S1 in Supplementary Material). Further analyses revealed that while the control and low dose groups significantly preferred their partners over strangers (*t* = 6.096, *p* < 0.00001 and *t* = 4.329, *p* < 0.0001, respectively), such preference was not seen in both the medium- and high-dose groups. In addition, male medium- and high-dose groups spent significantly less time in preferential contact with their partner than control, *t* = 2.856, *p* < 0.05 and *t* = 3.055, *p* < 0.05, respectively. For these two groups, the reduction in time spent in contact with the partner could not be explained by increases in time spent in the neutral compartment, *F* (3, 47) = 0.378, *p* = 0.769.

**Table 4:** Partner preference test statistics.

	Sex	Group	Partner contact	Stranger contact	Contact difference	Neutral zone
Partner preference 1	Males	Control	864.4 ± 187.6	409.1 ± 114.4	455.3 ± 265.0	2,756.5 ± 243.1
		Low	357.7 ± 235.2	545.3 ± 276.1	–187.7 ± 421.2	3,546.0 ± 428.8
		Medium	531.5 ± 264.9	887.0 ± 357.6	–355.5 ± 546.2	2,567.0 ± 290.4
		High	687.0 ± 262.3	472.2 ± 210.4	214.8 ± 422.5	2,978.6 ± 393.5
	Females	Control	704.7 ± 190.9	151.8 ± 91.5	552.9 ± 229.7	4,242.7 ± 431.7
		Low	767.5 ± 409.4	189.5 ± 118.2	578.0 ± 460.6	3,220.8 ± 526.8
		Medium	418.2 ± 179.5	472.6 ± 242.1	–54.4 ± 360.4	3,304.1 ± 392.7
		High	453.8 ± 233.8	724.7 ± 248.0	–270.9 ± 409.6	3,492.2 ± 527.7

Partner preference 2	Males	Control	1,931.8 ± 240.2	185.4 ± 79.8	1,746.3 ± 286.8	2,296.7 ± 197.1
		Low	2,107.1 ± 329.9	4.1 ± 2.7	2,103.0 ± 330.8	2,186.3 ± 383.9
		Medium	877.8 ± 339.9	617.8 ± 211.9	260.0 ± 491.5	2,445.7 ± 257.1
		High	712.0 ± 302.5	555.3 ± 233.1	156.7 ± 462.1	2750.5 ± 692.5
	Females	Control	1,921.0 ± 267.8	68.7 ± 53.4	1,855.4 ± 283.6	2,511.8 ± 266.4
		Low	2,084.6 ± 413.4	198.9 ± 198.9	1,905.6 ± 490.8	2,147.1 ± 311.2
		Medium	2,527.2 ± 270.2	0.0 ± 0.0	2,527.2 ± 270.2	1,706.9 ± 338.8
		High	1,856.8 ± 337.0	97.4 ± 97.4	1,770.2 ± 397.6	2,756.4 ± 307.9

*Values represent empirical means ± SEM. All variables are measured in seconds. Contact behaviors represent total time spent in social immobility (seconds), while neutral zone is the time spent in the neutral cage*.

Intranasal AVP did not affect partner contact in females during the first partner preference test and no group preferred the partner over the stranger. As for the second test, we did not find a significant effect of treatment on preferential mate choice. But while no differences existed between treatment groups, we did conduct *post hoc* analyses to confirm partner preference within each group. Unlike males in the second test, all female treatment groups did demonstrate a significant partner preference (control: *t* = 7.347, *p* < 0.00001; low: *t* = 4.755, *p* < 0.0001; med: *t* = 5.914, *p* < 0.00001, high: *t* = 4.127, *p* < 0.01) (Figure 4; Figure S1 in Supplementary Material). See Table 4 for all partner preference testing descriptive statistics.

**Figure 5:**
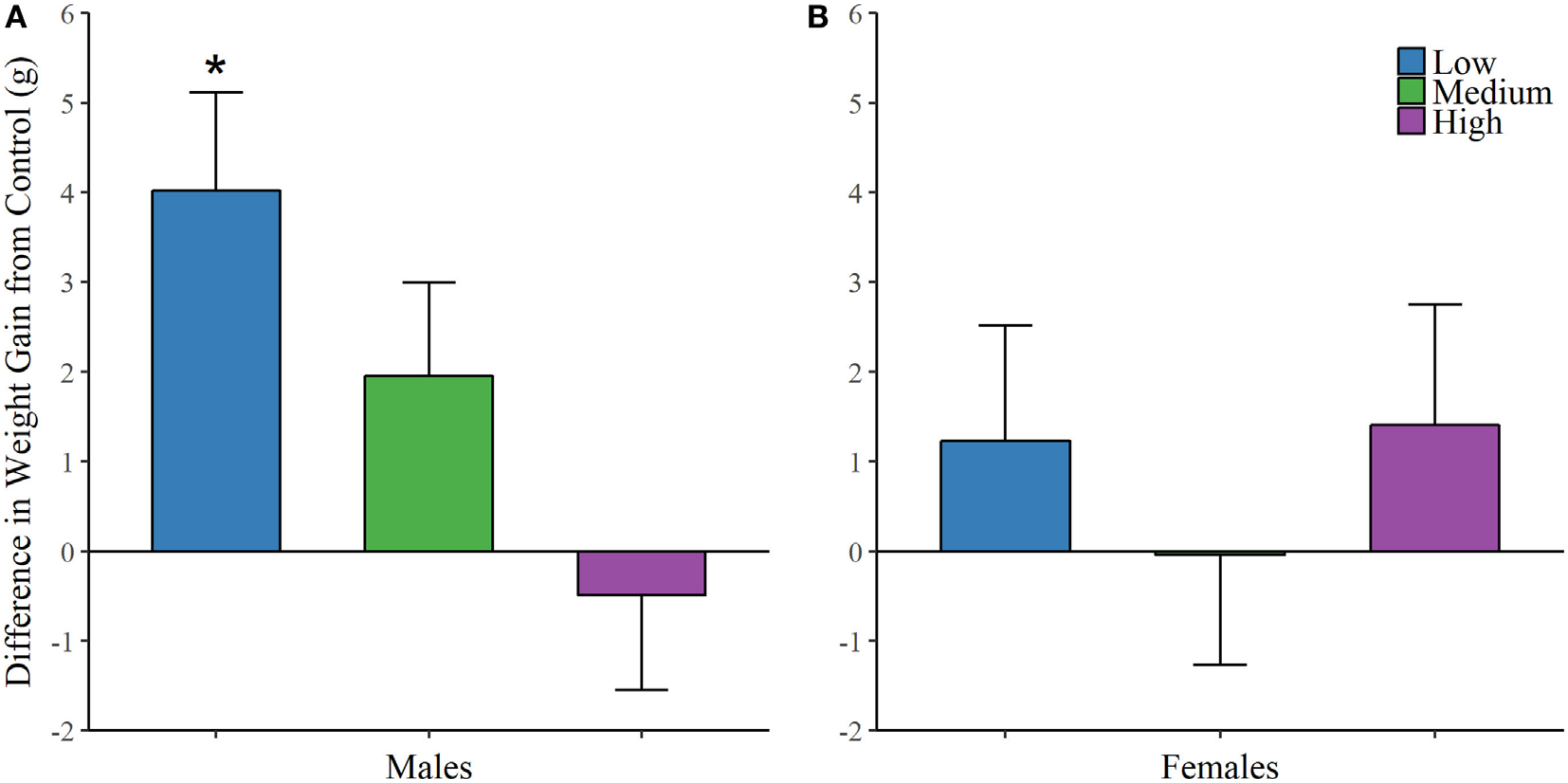
Early exposure to intranasal AVP (IN-AVP) increases weight gain in males. Values represent the difference in total weight gain across life from control (means + SE). The low-dose of IN-AVP increased weight gain across life in males **(A)** while no detectable effect was found in females **(B)**.

Finally, each animal was weighed on the first day of treatment and on the day of sacrifice. We found an effect of IN-AVP on weight change across life in males [*F* (3, 32.481) = 5.234, *p* < 0.01], but not in females; Figure 5. Further analysis revealed an increase in weight for low-dose males compared to control, *t* = 3.672, *p* < 0.01.

## Discussion

### Effects of IN-AVP Vary by Sex, Dose, and Context

We hypothesized that the effects of IN-AVP would (1) vary by dose, (2) be more prominent in males, and (3) exhibit contrasting effects depending on context. Our results confirm that IN-AVP modulates behavior in dose-specific ways. For example, the low dose increased weight gain, the medium dose increased fecal boli production, and the high dose increased aggression. We also found the most profound effects of IN-AVP administration in males, who exhibited both behavioral and physiological changes across life. Specifically, we found no effects of IN-AVP on adult female behavior, but males experienced impairments in partner preference formation and increases to aggression depending on the dose. We also detected context-specific contrasts in behavior. While IN-AVP did increase aggression during competitive encounters, we did not find increases in social behavior during non-threatening encounters. IN-AVP impaired sociability in males during partner preference testing without impacting sociability in juvenile affiliation or the first intrasexual aggression test (prior to partner preference). The changes to aggression experienced after partner preference testing also confirm that the effects of IN-AVP depend on context. This is further supported by the IN-AVP-stimulated increases in anxiety experienced by females in non-social contexts and increases to play behavior during social contexts (e.g., juvenile affiliation).

### Early IN-AVP Modulates Juvenile Anxiety and Sociality

In the present study, we found increases in fecal boli production across open field and novel object recognition testing for both males and females treated with the medium dose. Context-specific increases in fecal boli production have been associated with increased anxiety (43, 44); habituated animals produce successively fewer fecal boli with repeated testing (45). But, previous studies have also demonstrated a role for AVP in the regulation of gastric motility; systemic AVP injections increase gastric motility (46, 47) while AVP microinjections within the rat vagal nerve inhibit gastric motility (48). Given the fact that we did not find increases in other anxiety-related behaviors (e.g., freezing, autogrooming) alongside, the increases to fecal boli production, we suspect that peripheral AVP receptor effects on gastric motility may provide a stronger explanation for our findings. It would have been interesting to see if a more anxiogenic paradigm would have elicited a stronger response or if our results could be blocked by antidepressants.

The effects of IN-AVP during the juvenile period also appear to differ by context. While the treatment may potentially increase anxiety in both males and females during non-social novelty, we found evidence for increases in sociality with a novel social partner. Specifically, the medium dose selectively increased play bouts in females (with no increase in males). This may reflect slight differences in the quantity of play exhibited by male and female prairie voles; male control voles engaged in marginally higher bouts of play than female control voles. Alternatively, it is also possible that females may be more susceptible to the effects of AVP in play behavior since male prairie voles have more AVP-containing neurons than females in several neural regions, such as the medial amygdala and bed nucleus of the stria terminalis (22).

In rodents, the AVP system seems to regulate social play differently between males and females. For example, intracerebroventricular (ICV) administration of AVPR1a antagonists in rats increased social play in females and decreased it in males (49). However, site-specific injections of AVPR1a into the lateral septum produced the opposite results, increasing play in males while reducing it in females (6). Without measuring the effect of chronic IN-AVP on regulation of AVP receptors and peptide throughout the brain, it is difficult to determine the neural mechanism for our findings that medium-dose IN-AVP increased play bouts in females. Repeated activation of AVPR1a has been shown to cause internalization, decreasing the membrane density of AVPR1a (50). It is possible that the administration period and frequency implemented in this study was sufficient to decrease AVPR1a densities across the brain, approximating the effect of ICV AVPR1a antagonist administration as shown by Veenema et al. (49). But, if downregulation of AVPR1a is the cause of our observed effects, it is curious that we did not see a simultaneous decrease in play activity for males treated with IN-AVP. Regardless, we might expect the effects of intranasally administered AVP in prairie voles to differ from other more direct delivery routes (e.g., microinjections) and from other rodent species, especially, since the mechanism for behavioral effects of intranasal delivery has not yet been confirmed (51).

### Early IN-AVP Modulates Pair-Bonding Behavior Later in Life

Early studies suggested that OT activity on the OTR was more important for pair-bond formation in females, while AVP activity on the AVPR1a was more important in males (52–55). More recent studies have produced more subtlety than this strict dichotomy, suggesting instead that OT and AVP are involved in partner preference formation in both sexes (56–59). Cho et al. (57) found that ICV administration of OT or AVP could facilitate pair-bond formation in both males and females while concurrent administration of either OT receptor or AVPR1a antagonists blocked this effect. In our study, we demonstrated that a single week of twice daily exposure to IN-AVP during the early juvenile stage can disrupt pair-bond formation later in life. These effects were sex-dependent, occurring only in the male medium- and high-dose groups, and did not reflect changes in the amount of time spent in the neutral cage. On the contrary, all experimental groups in females successfully demonstrated a partner preference by the time of the second test.

Previous studies have demonstrated that pair-bond behavior in prairie voles is highly susceptible to manipulation of the AVP system. In adult prairie voles, site-specific AVPR1a antagonists within the ventral pallidum prevented pair-bond formation (60), while selectively increasing AVPR1a densities in the ventral forebrain facilitated pair-bond formation (61). Overexpression of AVPR1a within the ventral pallidum (60) and ventral forebrain (62) of the promiscuous meadow vole substantially increased partner preference behavior. As mentioned before, repeated activation of AVPR1a can lead to physiological tolerance (50). Since the observed chronic effects of IN-AVP administration in our study approximate the effects of acute AVPR1a antagonist administration in other studies, we suspect that a single week of twice daily exposure to AVP should be sufficient to decrease AVPR1a densities in specific neural regions, which are critical for pair-bond formation in males. Complementary work from our lab has shown that chronic OT exposure also impairs partner preference formation in prairie voles (63) with subsequent changes to OTR receptor and AVP peptide concentrations (unpublished data).

Given our results, we suspect that the physiological mechanisms behind the disrupted partner preference in males may differ between the medium- and high-dose groups. Specifically, the high dose also resulted in a substantial increase in aggressive behavior during the second intrasexual aggression test, which was conducted approximately 24 h following partner preference testing. These findings are similar to those found by Stribley and Carter (7); early postnatal exposure to the high dose of AVP increased aggression in sexually naïve prairie voles compared to control. In prairie voles, aggressive tendencies naturally increase following induction of the pair-bond. Gobrogge et al. (64) showed that 2 weeks of cohabitation with a female intensely increased male aggression toward both novel male and female conspecifics; these males maintained elevated levels of social affiliation with their female partners during this time. But, the increase in aggression experienced by high-dose males in our study occurred in the absence of a preference for a female partner.

Outside of the pair-bond, AVP modulates aggressive behavior in sex-specific ways. AVP injections within the anterior hypothalamus stimulate aggression in male Syrian hamsters but inhibits it in females (65, 66). Also, male prairie voles who received low amounts of parental handling early in life engaged in more aggression in adulthood (67). Given the stimulatory effects of AVP in males on aggression and the lack of pair-bond formation in high-dose males, the increases in aggression is likely unaffiliated with mate-guarding. On the contrary, there is also the potential for delayed pair-bond formation in the high-dose males. While this group may not have formed a partner preference at 24 h, they may have formed it at some point between the end of the second partner preference and the second intrasexual aggression test (which was an additional 24 h). Thus, it is possible that high-dose males experienced a delay in partner preference formation, but had an exacerbated mate-guarding response once the pair-bond occurred. Regardless, the medium and high doses may have different effects on AVPR1a in brain regions involved in pair bonding versus aggression.

We also found that the low-dose males gained significantly more weight than control. Inappropriate AVP secretion to the periphery (68) and the use of synthetic AVP (desmopressin) has been linked to weight gain in humans (69). Though we reported only the results of weight gain across life, we also measured the difference in weight gain across the dosing period. No significant difference in weight gain was found for any of the treatment groups during this time, but there was a suggestive increase in weight gain for the low-dose group in males (Cohen’s *d* = 0.66). As supported by this experiment, early life manipulations can change adult behavior (and likely physiology). Therefore, it is possible that the slight (statistically insignificant) changes in weight gain for males treated with the low dose of IN-AVP across the dosing period were subsequently exacerbated across life.

### Limitations

Caveats in interpretation of these results are the lack of any animal model for autism with clear constructive validity (70, 71), and the variability of OT and AVP receptors across different taxa and species. This variability may lead to differences in responses to these neuropeptides across species. In a previous study, we found that chronic OT impaired pair bonding in male prairie voles at certain doses (63). However, the same dose did not change mouse social behavior, either in BTBR mice (a rodent model of reduced sociability) or in their strain control (72). In our quest to translate neuropeptide results from animal models, we should consider the neurobiology and natural history of the animal model, as well as the dosage, sex, context of administration, and other testing conditions. Future studies in animal models and in humans will reveal which model is most predictive.

Another limitation is the potential for the confounding effects of repeated behavioral testing on the results. Our study employs several behavioral paradigms in the early juvenile period as well as adulthood. It is possible that this combination of testing could have obscured or attenuated treatment and sex effects of IN-AVP, particularly given the density of testing and the frequency of experimental handling early in life. But as mentioned previously, the increases in aggression experienced by the high-dose group in adulthood do replicate the results of Stribley and Carter (7) who did not employ the same intensity of behavioral testing.

## Conclusion

The results of this study confirm that the contextually pro-social effects of IN-AVP administration may differ from the long-term effects of a developmental exposure. Specifically, the impairment of partner preference displayed by male prairie voles in our study is notably opposite of the acute, facilitatory effects of AVP administration on partner preference formation (55) and social contact (73, 74) demonstrated in other studies. Further studies exploring differences in developmental timing and varied dosing schedules will contribute to our understanding of the AVP system while potentially informing clinical pursuits.

## Ethics Statement

This study and protocol were reviewed and approved by the Institutional Animal Care and Use Committee (IACUC) at the University of California, Davis, and complied with National Institutes of Health ethical guidelines as set forth in the Guide for Lab Animal Care.

## Author Contributions

TS planned and executed all aspects of this work under the direction of KB. JB, JD, SY, and JV helped provide intranasal treatments, behavioral testing, and video scoring. JT scored video. All authors participated in writing and editing this article.

## Conflict of Interest Statement

The authors declare that the research was conducted in the absence of any commercial or financial relationships that could be construed as a potential conflict of interest.

## References

[1] Caldwell HK Albers HE. Oxytocin, vasopressin, and the motivational forces that drive social behaviors. Curr Top Behav Neurosci (2016) 27:51–103. 10.1007/7854_2015_390 26472550

[2] Baribeau DA Anagnostou E. Oxytocin and vasopressin: linking pituitary neuropeptides and their receptors to social neurocircuits. Front Neurosci (2015) 9:335. 10.3389/fnins.2015.00335 26441508 PMC4585313

[3] Carter CS DeVries AC Taymans SE Roberts RL Williams JR Getz LL Peptides, steroids, and pair bonding. Ann N Y Acad Sci (1997) 807:260–72. 10.1111/j.1749-6632.1997.tb51925.x 9071356

[4] Wang Z Young LJ Liu Y Insel TR. Species differences in vasopressin receptor binding are evident early in development: comparative anatomic studies in prairie and montane voles. J Comp Neurol (1997) 378(4):535–46. 10.1002/(SICI)1096-9861(19970224)378:4<535::AID-CNE8>3.0.CO;2-3 9034909

[5] Varlinskaya EI Petrov ES Robinson SR Smotherman WP. Behavioral effects of centrally administered arginine vasopressin in the rat fetus. Behav Neurosci (1994) 108(2):395–409. 10.1037/0735-7044.108.2.395 8037883

[6] Bredewold R Smith CJ Dumais KM Veenema AH. Sex-specific modulation of juvenile social play behavior by vasopressin and oxytocin depends on social context. Front Behav Neurosci (2014) 8:216. 10.3389/fnbeh.2014.00216 24982623 PMC4058593

[7] Stribley JM Carter CS. Developmental exposure to vasopressin increases aggression in adult prairie voles. Proc Natl Acad Sci U S A (1999) 96(22):12601–4. 10.1073/pnas.96.22.12601 10535968 PMC23008

[8] Baran NM Tomaszycki ML Adkins-Regan E. Early life manipulations of the nonapeptide system alter pair maintenance behaviors and neural activity in adult male zebra finches. Front Behav Neurosci (2016) 10:58. 10.3389/fnbeh.2016.00058 27065824 PMC4810809

[9] Carson DS Garner JP Hyde SA Libove RA Berquist SW Hornbeak KB Arginine vasopressin is a blood-based biomarker of social functioning in children with autism. PLoS One (2015) 10(7):e0132224. 10.1371/journal.pone.0132224 26200852 PMC4511760

[10] Miller M Bales KL Taylor SL Yoon J Hostetler CM Carter CS Oxytocin and vasopressin in children and adolescents with autism spectrum disorders: sex differences and associations with symptoms. Autism Res (2013) 6(2):91–102. 10.1002/aur.1270 23413037 PMC3657571

[11] Xu XJ Shou XJ Li J Jia MX Zhang JS Guo Y Mothers of autistic children: lower plasma levels of oxytocin and Arg-vasopressin and a higher level of testosterone. PLoS One (2013) 8(9):e74849. 10.1371/journal.pone.0074849 24086383 PMC3783493

[12] Quintana DS Westlye LT Smerud KT Mahmoud RA Djupesland PG Andreassen OA. Reliability of basal plasma vasopressin concentrations in healthy male adults. Acta Neuropsychiatr (2016):1–7. 10.1017/neu.2016.67 27923411

[13] Zhang R Zhang HF Han JS Han SP Genes related to oxytocin and arginine-vasopressin pathways: associations with autism spectrum disorders. Neurosci Bull (2017) 33:238–46. 10.1007/s12264-017-0120-7 28283809 PMC5360847

[14] Brunnlieb C Nave G Camerer CF Schosser S Vogt B Munte TF Vasopressin increases human risky cooperative behavior. Proc Natl Acad Sci U S A (2016) 113(8):2051–6. 10.1073/pnas.1518825113 26858433 PMC4776476

[15] Gozzi M Dashow EM Thurm A Swedo SE Zink CF Effects of oxytocin and vasopressin on preferential brain responses to negative social feedback. Neuropsychopharmacology (2017) 42:1409–19. 10.1038/npp.2016.248 27796303 PMC5436111

[16] Guastella AJ Kenyon AR Alvares GA Carson DS Hickie IB. Intranasal arginine vasopressin enhances the encoding of happy and angry faces in humans. Biol Psychiatry (2010) 67(12):1220–2. 10.1016/j.biopsych.2010.03.014 20447617

[17] Belyakova AS Sinjushin AA Voskresenskaya OG Kamensky AA Golubovich VP The analog of arginine-vasopressin (6-9) fragment, Ac-D-SPRG, exhibits antidepressant action in rats in case of intranasal injection. Neurochem J (2015) 9(3):201–5. 10.1134/S1819712415030034

[18] Jarcho MR Mendoza SP Mason WA Yang X Bales KL. Intranasal vasopressin affects pair bonding and peripheral gene expression in male *Callicebus cupreus*. Genes Brain Behav (2011) 10(3):375–83. 10.1111/j.1601-183X.2010.00677.x 21255269 PMC3086990

[19] Hammock EA Young LJ. Oxytocin, vasopressin and pair bonding: implications for autism. Philos Trans R Soc Lond B Biol Sci (2006) 361(1476):2187–98. 10.1098/rstb.2006.1939 17118932 PMC1764849

[20] Insel TR O’Brien DJ Leckman JF. Oxytocin, vasopressin, and autism: is there a connection? Biol Psychiatry (1999) 45(2):145–57. 10.1016/s0006-3223(98)00142-5 9951561

[21] Bamshad M Novak MA De Vries GJ. Sex and species differences in the vasopressin innervation of sexually naive and parental prairie voles, *Microtus ochrogaster* and meadow voles, *Microtus pennsylvanicus*. J Neuroendocrinol (1993) 5(3):247–55. 10.1111/j.1365-2826.1993.tb00480.x 8319000

[22] Wang Z Smith W Major DE De Vries GJ Sex and species differences in the effects of cohabitation on vasopressin messenger RNA expression in the bed nucleus of the stria terminalis in prairie voles (*Microtus ochrogaster*) and meadow voles (*Microtus pennsylvanicus*). Brain Res (1994) 650(2):212–8. 10.1016/0006-8993(94)91784-1 7953686

[23] Kelly AM Ophir AG. Compared to what: what can we say about nonapeptide function and social behavior without a frame of reference? Curr Opin Behav Sci (2015) 6:97–103. 10.1016/j.cobeha.2015.10.010 26858966 PMC4742393

[24] Chau MJ Stone AI Mendoza SP Bales KL Is play behavior sexually dimorphic in monogamous species? Ethology (2008) 114(10):989–98. 10.1111/j.1439-0310.2008.01543.x

[25] Williams JR Catania KC Carter CS. Development of partner preferences in female prairie voles (*Microtus ochrogaster*): the role of social and sexual experience. Horm Behav (1992) 26(3):339–49. 10.1016/0018-506X(92)90004-F 1398553

[26] DeVries AC Carter CS Sex differences in temporal parameters of partner preference in prairie voles (*Microtus ochrogaster*). Can J Zool (1999) 77(6):885–9. 10.1139/cjz-77-6-885

[27] Core Team R. R: A Language and Environment for Statistical Computing. Vienna, Austria: R Foundation for Statistical Computing (2016).

[28] Scheipl F Greven S Kuechenhoff H Size and power of tests for a zero random effect variance or polynomial regression in additive and linear mixed models. Comput Stat Data Anal (2008) 52(7):3283–99. 10.1016/j.csda.2007.10.022

[29] Crainiceanu CM Ruppert D Likelihood ratio tests in linear mixed models with one variance component. J R Stat Soc Series B Stat Methodol (2004) 66(1):165–85. 10.1111/j.1467-9868.2004.00438.x

[30] Bates D Mächler M Bolker B Walker S Fitting Linear Mixed-Effects Models Using lme4. J Stat Softw (2015) 67(1):1–48. 10.18637/jss.v067.i01

[31] Pleil JD. QQ-plots for assessing distributions of biomarker measurements and generating defensible summary statistics. J Breath Res (2016) 10(3):035001. 10.1088/1752-7155/10/3/035001 27491525

[32] Razali NM Wah YB Power comparisons of Shapiro-Wilk, Kolmogorov-Smirnov, Lilliefors and Anderson-Darling tests. J Stat Model Anal (2011) 2(1):21–33.

[33] Field A Discovering Statistics Using IBM SPSS Statistics. SAGE (2013).

[34] George D SPSS for Windows Step by Step: A Simple Study Guide and Reference, 17.0 Update, 10/e. India: Pearson Education (2011).

[35] Gravetter FJ Wallnau LB Forzano L-AB Essentials of Statistics for the Behavioral Sciences. Nelson Education (2016).

[36] Lerman G McCoy M Tropp JA Zhang T Robust Computation of Linear Models, or How to Find a Needle in a Haystack. arXiv e-prints (2012).

[37] McKean JW Robust analysis of linear models. Stat Sci (2004) 19:562–70. 10.1214/088342304000000549

[38] Venables WN Ripley BD Modern Applied Statistics with S. New York: Springer (2002).

[39] Koller M robustlmm: an R package for robust estimation of linear mixed-effects models. J Stat Softw (2016) 75(6):1–24. 10.18637/jss.v075.i06 PMC7351245 32655332

[40] Fox J Weisberg S An R Companion to Applied Regression. Thousand Oaks, CA: SAGE (2011).

[41] Lenth RV Least-squares means: the R package lsmeans. J Stat Softw (2016) 69(1):1–33. 10.18637/jss.v069.i01

[42] Dunnett CW New tables for multiple comparisons with a control. Biometrics (1964) 20(3):482–91. 10.2307/2528490

[43] Mönnikes H Schmidt BG Taché Y. Psychological stress-induced accelerated colonic transit in rats involves hypothalamic corticotropin-releasing factor. Gastroenterology (1993) 104(3):716–23. 10.1016/0016-5085(93)91006-4 8440432

[44] Williams CL Peterson JM Villar RG Burks TF. Corticotropin-releasing factor directly mediates colonic responses to stress. Am J Physiol (1987) 253(4):G582. 2821826 10.1152/ajpgi.1987.253.4.G582

[45] Zhang W Hetzel A Shah B Atchley D Blume SR Padival MA Greater physiological and behavioral effects of interrupted stress pattern compared to daily restraint stress in rats. PLoS One (2014) 9(7):e102247. 10.1371/journal.pone.0102247 25014526 PMC4094544

[46] Li L Kong X Liu H Liu C. Systemic oxytocin and vasopressin excite gastrointestinal motility through oxytocin receptor in rabbits. Neurogastroenterol Motil (2007) 19(10):839–44. 10.1111/j.1365-2982.2007.00953.x 17883435

[47] Qin J Liu K Wang PS Liu C. V1 receptor in ENS mediates the excitatory effect of vasopressin on circular muscle strips of gastric body in vitro in rats. Regul Pept (2009) 157(1–3):32–6. 10.1016/j.regpep.2009.06.003 19523991

[48] Zhu J Chang L Xie J Ai H. Arginine vasopressin injected into the dorsal motor nucleus of the vagus inhibits gastric motility in rats. Gastroenterol Res Pract (2016) 2016:4618672. 10.1155/2016/4618672 26843857 PMC4710933

[49] Veenema AH Bredewold R De Vries GJ. Sex-specific modulation of juvenile social play by vasopressin. Psychoneuroendocrinology (2013) 38(11):2554–61. 10.1016/j.psyneuen.2013.06.002 23838102 PMC3812261

[50] Terrillon S Cheng LL Stoev S Mouillac B Barberis C Manning M Synthesis and characterization of fluorescent antagonists and agonists for human oxytocin and vasopressin V1a receptors. J Med Chem (2002) 45(12):2579–88. 10.1021/jm010526+ 12036367

[51] Quintana DS Alvares GA Hickie IB Guastella AJ. Do delivery routes of intranasally administered oxytocin account for observed effects on social cognition and behavior? A two-level model. Neurosci Biobehav Rev (2015) 49:182–92. 10.1016/j.neubiorev.2014.12.011 25526824

[52] Insel TR Hulihan TJ. A gender-specific mechanism for pair bonding: oxytocin and partner preference formation in monogamous voles. Behav Neurosci (1995) 109(4):782–9. 10.1037/0735-7044.109.4.782 7576222

[53] Lim MM Hammock EA Young LJ The role of vasopressin in the genetic and neural regulation of monogamy. J Neuroendocrinol (2004) 16(4):325–32. 10.1111/j.0953-8194.2004.01162.x 15089970

[54] Nair HP Young LJ. Vasopressin and pair-bond formation: genes to brain to behavior. Physiology (Bethesda) (2006) 21:146–52. 10.1152/physiol.00049.2005 16565480

[55] Winslow JT Hastings N Carter CS Harbaugh CR Insel TR. A role for central vasopressin in pair bonding in monogamous prairie voles. Nature (1993) 365(6446):545–8. 10.1038/365545a0 8413608

[56] Aragona BJ Wang Z. Dopamine regulation of social choice in a monogamous rodent species. Front Behav Neurosci (2009) 3:15. 10.3389/neuro.08.015.2009 19707518 PMC2729670

[57] Cho MM DeVries AC Williams JR Carter CS. The effects of oxytocin and vasopressin on partner preferences in male and female prairie voles (*Microtus ochrogaster*). Behav Neurosci (1999) 113(5):1071–9. 10.1037/0735-7044.113.5.1071 10571489

[58] Ophir AG Gessel A Zheng DJ Phelps SM. Oxytocin receptor density is associated with male mating tactics and social monogamy. Horm Behav (2012) 61(3):445–53. 10.1016/j.yhbeh.2012.01.007 22285648 PMC3312950

[59] Wang H Duclot F Liu Y Wang Z Kabbaj M. Histone deacetylase inhibitors facilitate partner preference formation in female prairie voles. Nat Neurosci (2013) 16(7):919–24. 10.1038/nn.3420 23727821 PMC3703824

[60] Lim MM Young LJ. Vasopressin-dependent neural circuits underlying pair bond formation in the monogamous prairie vole. Neuroscience (2004) 125(1):35–45. 10.1016/j.neuroscience.2003.12.008 15051143

[61] Pitkow LJ Sharer CA Ren XL Insel TR Terwilliger EF Young LJ. Facilitation of affiliation and pair-bond formation by vasopressin receptor gene transfer into the ventral forebrain of a monogamous vole. J Neurosci (2001) 21(18):7392–6. 11549749 10.1523/JNEUROSCI.21-18-07392.2001 PMC6762997

[62] Lim MM Wang Z Olazabal DE Ren X Terwilliger EF Young LJ Enhanced partner preference in a promiscuous species by manipulating the expression of a single gene. Nature (2004) 429(6993):754–7. 10.1038/nature02539 15201909

[63] Bales KL Perkeybile AM Conley OG Lee MH Guoynes CD Downing GM Chronic intranasal oxytocin causes long-term impairments in partner preference formation in male prairie voles. Biol Psychiatry (2013) 74(3):180–8. 10.1016/j.biopsych.2012.08.025 23079235 PMC3556198

[64] Gobrogge KL Liu Y Jia X Wang Z. Anterior hypothalamic neural activation and neurochemical associations with aggression in pair-bonded male prairie voles. J Comp Neurol (2007) 502(6):1109–22. 10.1002/cne.21364 17444499

[65] Ferris CF Melloni RH Jr Koppel G Perry KW Fuller RW Delville Y. Vasopressin/serotonin interactions in the anterior hypothalamus control aggressive behavior in golden hamsters. J Neurosci (1997) 17(11):4331–40. 9151749 10.1523/JNEUROSCI.17-11-04331.1997 PMC6573530

[66] Gutzler SJ Karom M Erwin WD Albers HE Arginine-vasopressin and the regulation of aggression in female Syrian hamsters (*Mesocricetus auratus*). Eur J Neurosci (2010) 31(9):1655–63. 10.1111/j.1460-9568.2010.07190.x 20525078

[67] Perkeybile AM Bales KL. Early rearing experience is related to altered aggression and vasopressin production following chronic social isolation in the prairie vole. Behav Brain Res (2015) 283:37–46. 10.1016/j.bbr.2015.01.025 25623420 PMC4351180

[68] Khan I Zimmerman B Brophy P Kamath S. Masking of syndrome of inappropriate antidiuretic hormone secretion: the isonatremic syndrome. J Pediatr (2014) 165(4):722–6. 10.1016/j.jpeds.2014.05.051 24996987

[69] Rembratt A Riis A Norgaard JP. Desmopressin treatment in nocturia; an analysis of risk factors for hyponatremia. Neurourol Urodyn (2006) 25(2):105–9. 10.1002/nau.20168 16304673

[70] Bey AL Jiang YH Overview of mouse models of autism spectrum disorders. Curr Protoc Pharmacol (2014) 66:5.66.1–26. 10.1002/0471141755.ph0566s66 25181011 PMC4186887

[71] Kazdoba TM Leach PT Yang M Silverman JL Solomon M Crawley JN. Translational mouse models of autism: advancing toward pharmacological therapeutics. Curr Top Behav Neurosci (2016) 28:1–52. 10.1007/7854_2015_5003 27305922 PMC5116923

[72] Bales KL Solomon M Jacob S Crawley JN Silverman JL Larke RH Long-term exposure to intranasal oxytocin in a mouse autism model. Transl Psychiatry (2014) 4:e480. 10.1038/tp.2014.117 25386957 PMC4259989

[73] Ramos L Hicks C Kevin R Caminer A Narlawar R Kassiou M Acute prosocial effects of oxytocin and vasopressin when given alone or in combination with 3,4-methylenedioxymethamphetamine in rats: involvement of the V1A receptor. Neuropsychopharmacology (2013) 38(11):2249–59. 10.1038/npp.2013.125 23676791 PMC3773675

[74] Ramos L Hicks C Caminer A McGregor IS. Inhaled vasopressin increases sociability and reduces body temperature and heart rate in rats. Psychoneuroendocrinology (2014) 46:46–51. 10.1016/j.psyneuen.2014.04.013 24882157

