# Supplementary material for: Early Intranasal Vasopressin Administration Impairs Partner Preference in Adult Male Prairie Voles (Microtus ochrogaster)
Source: Front Endocrinol (Lausanne). 2017 Jun 28;8:145. doi: 10.3389/fendo.2017.00145 (PMC5487415; doi:10.3389/fendo.2017.00145)
Supplement: Supplementary file 1 [file Data_Sheet_1.DOCX]

Supplementary Material

Early Intranasal Vasopressin Administration Impairs Partner Preference in Adult Male Prairie Voles (Microtus ochrogaster)

Trenton C. Simmons*, Jessica F. Balland, Janeet Dhauna, Sang Yun Yang, Jason L. Traina, Jessica Vazquez, Karen L. Bales

*** Correspondence:** Trenton C. Simmons: simmons.tc@outlook.com

# Supplementary Figures


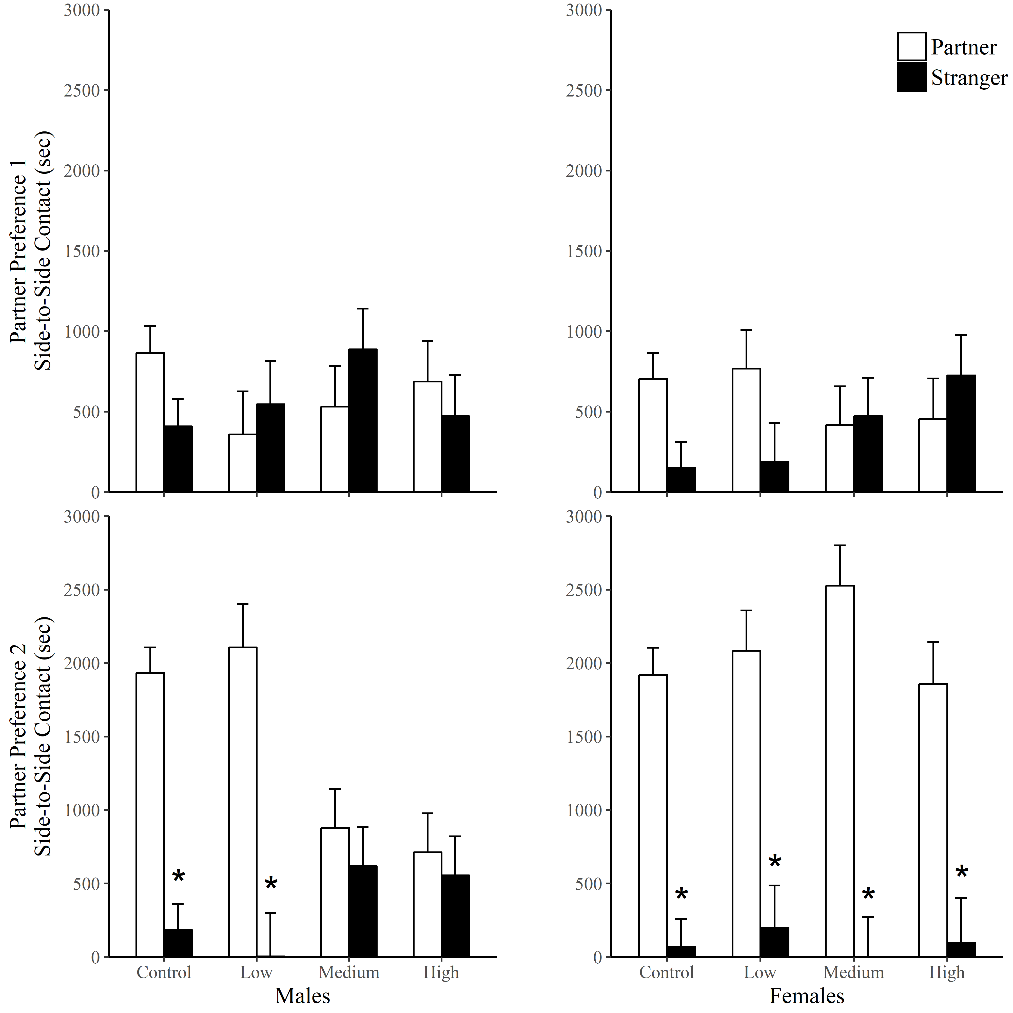


**Supplementary Figure 1.** Early exposure to IN-AVP blocks partner preference formation in males. Values represent group means + standard error. During the first partner preference test, subjects were housed with potential mates for an insufficient amount of time to form a preference (upper row). IN-AVP did not facilitate partner preference in either males (A) or females (B) during this test. The second partner preference test was completed following 24 hours of cohabitation between each test subject and their respective partners from the first test (bottom row). The medium and high-doses of IN-AVP shunted partner preference in males (C) but all female treatment groups (D) successfully preferred the partner over the stranger.
